# Brain Natriuretic Peptide Production and Secretion in Inflammation

**DOI:** 10.1155/2012/962347

**Published:** 2012-11-28

**Authors:** Tsuneo Ogawa, Adolfo J. de Bold

**Affiliations:** Cardiovascular Endocrinology Laboratory, University of Ottawa Heart Institute, 40 Ruskin Street, Ottawa, ON, Canada K1Y 4W7

## Abstract

Gene expression and secretion of the cardiac polypeptide hormones atrial natriuretic factor (ANF) and brain natriuretic peptide (BNP) are simultaneously upregulated in various cardiac disorders such as congestive heart failure, ischemic heart disease, and hypertensive heart disease, in which hemodynamic or neuroendocrine changes are key components in the progression of disease. However, during acute cardiac allograft rejection, plasma BNP levels are increased but not those of ANF. Successful treatment of the rejection episode decreases the elevated plasma BNP to prerejection values suggesting that substances related to inflammation may selectively influence BNP gene expression. Indeed, cytokines such as TNF**α** and IL-1**β** selectively stimulate cardiac BNP at the transcriptional and translational levels in cardiomyocyte cultures without affecting ANF. This selective BNP increase is seen *in vivo*, in addition to acute cardiac allograft rejection, in several circumstances where inflammation significantly contributes to the pathogenesis of disease such as in sepsis and in acute myocarditis.

## 1. Introduction

Seven years after the discovery of the natriuretic factor from the atria of the heart (ANF) [1, 2] and five years after the first purification and sequencing of its circulating form [3], brain natriuretic peptide (BNP) was isolated from porcine brain [4]. Subsequently, BNP was found to be most abundantly expressed in the atria of the heart. The biologically active, circulating forms of these cardiac natriuretic peptides (NPs) ANF (ANF_99–126_) and BNP (BNP_77–108_) are C-terminal fragments from their respective prohormones: ANF_1–126_ and BNP_1–108_. The N-terminal fragments of ANF (NT-proANF) and BNP (NT-proBNP) coexist in blood with the C-terminal fragments. Measurement of both N- and C-terminal peptides in blood has been used in clinical studies in relation to cardiovascular disorders [5].

ANF and BNP share most biological properties including diuretic, natriuretic, vasorelaxant, and cardiac antihypertrophic and antifibrotic properties that are effected by signaling through a common receptor: the guanylyl cyclase- (GC-) A receptor [6]. However, some differences between ANF and BNP have been reported on control of gene expression and phenotype arising from observations in transgenic animals. BNP gene expression was induced more rapidly than that of ANF in the left ventricles following coronary artery ligation in rats [7]. Mice with genetically reduced production of ANF can lead to salt-sensitive hypertension [8], but mice with targeted disruption of the BNP gene develop multifocal fibrotic lesions in the ventricles without systemic hypertension or ventricular hypertrophy [9].

Biological assays leading to the discovery of ANF showed that extracts of ventricular tissue do not possess the natriuretic, diuretic, or hypotensive activity of atrial tissue extracts [1] suggesting that both ANF and BNP were solely produced in the atria. The advent of the more sensitive radioimmunoassay techniques to measure these NPs revealed that they were also present in ventricular muscle albeit at much lower concentrations than in the atria. Transcript abundance reflects this difference [5, 10, 11]. In addition, in both atria and ventricles, ANF is always more abundant than BNP [12, 13]. These facts directly contradict the notion found sometimes in the recent literature that BNP is a ventricular hormone while ANF is an atrial hormone.

The release of ANF and BNP from the heart into the blood leads to circulating levels that reflect their relative abundance in cardiac tissue. In the basal state, human plasma levels for ANF and BNP are 5.2 fmol/mL and 2.5 fmol/mL [14], respectively, while in rats the levels are 25 fmol/mL and 2.5 fmol/mL, respectively [12].

The neurohumoral and hemodynamic changes associated with pathologies impacting on the heart such as chronic congestive heart failure elevate NP circulating levels. For example, blood samples from patients who were in end-stage heart failure before transplantation had C-terminal ANF and C-terminal BNP plasma levels of 356 ± 21 pg/mL (*n* = 77) and 259 ± 37 pg/mL (*n* = 76) (mean ± SEM) [5], respectively. Interestingly, replacement of the failing ventricle as it is done in orthotropic heart transplantation does not result in normalization of either BNP or ANF plasma levels even after normalization of intracardiac pressures and the renin angiotensin aldosterone system occurs [15–18]. This finding argues against an important ventricular contribution to circulating NP levels.

The measurement of plasma NP levels has become useful diagnostic and prognostic tools in various cardiovascular diseases such as heart failure, myocardial infarction, and acute coronary syndrome (for a review, see [5]). In studies comparing the diagnostic value for detecting systolic or diastolic heart failure or for assessing mortality in patients with chronic congestive heart failure, BNP appeared to fare better as a biomarker than ANF [19–23]. Following these observations, BNP or NT-proBNP has been used most commonly as diagnostic or prognostic biomarkers for a variety of cardiac disorders including myocardial remodeling [24], asymptomatic left ventricular dysfunction [25], sudden cardiac death and ventricular arrhythmia [26], and acute pulmonary embolism [27]. Although there are subtle differences between ANF and BNP, the stimuli for production and secretion of these two hormones have been considered almost identical until a discoordinated change of ANF and BNP was revealed in allograft rejection episodes as described in the following.

## 2. Plasma BNP in Acute Cardiac Allograft Rejection

While investigating changes in NP plasma levels in human cardiac transplantation, we found that, during an acute rejection episode, BNP but not ANF increased significantly over their levels determined prior to the rejection episode [16]. Successful treatment of the rejection episode resulted in decrease of BNP plasma levels to values observed prior to acute rejection.

In a study of 69 consecutive allograft cardiac recipients, we found that plasma BNP and NT-proBNP levels in patients with endomyocardial biopsies graded 3 according to the International Society of Heart and Lung Transplantation (ISHLT) were significantly higher than those in ISHLT grade 0 to 2. Plasma ANF did not change with increasing ISHLT grade [17]. Others also have reported more recently an association between plasma BNP and cardiac cellular rejection [28–31]. Similar findings were seen in pediatric cardiac allograft recipients [32, 33]. Finally, plasma BNP or NT-proBNP levels in postcardiac transplantation strongly and independently predicted cardiac mortality [34, 35].

Other studies have not found a significant relationship between plasma BNP and endomyocardial biopsy rejection grade. No significant differences were seen in the biopsy score between high BNP group (BNP > 150 pg/mL) and low BNP group (BNP < 150 pg/mL) [36]. In another study, the rejection history based on ISHLT grade classification was not independently correlated with plasma BNP levels [37]. Plasma BNP levels were found not to differ according to the presence or degree of histological rejection [38, 39]. Plasma BNP levels were significantly higher in patients with ISHLT grade 2 or higher compared to nonrejecting recipients within three months after transplant. However, after the third month, BNP levels were similar in patients with and without rejection [40]. Some of these investigations found an association with pulmonary artery pressure or pulmonary capillary wedge pressure [36–38, 40]. The increase in pulmonary pressure may lead to a variable degree of tricuspid valve insufficiency resulting in right ventricular failure hence increasing BNP gene expression and plasma BNP levels. Thus, these changes may disturb the association between plasma BNP and rejection. Other factors such as time posttransplant or renal function may also affect plasma BNP levels possibly disturbing the association between BNP and rejection [37].

In an investigation on the relationship between BNP levels and hemodynamic change in rejecting and nonrejecting patients, we reported that cardiac filling pressures were correlated with plasma BNP or NT-proBNP only in ISHLT grade 0 and 1 cases and that this correlation did not exist for ISHLT grade greater than 2 [17]. This finding suggests that, in the latter category, BNP plasma levels are determined for factors other than or in addition to the hemodynamic status of the patient. In multivariable linear regression studies examining the outcome of increasing seriousness of rejection, BNP predicted a new episode of rejection independent of hemodynamic measurement [31]. These results suggest that the elevation of BNP during allograft rejection episode is not the result of hemodynamic change.

It is important to note that in our investigations [16] the increase in BNP plasma levels observed during acute cardiac allograft rejection was evident when comparing the individual patient values with their plasma BNP baseline value, that is on the week prior to rejection. That is to say that a threshold absolute value for BNP plasma levels indicative of acute rejection could not be established across the patient population. We also observed that BNP plasma values tended to be elevated in the sample prior to the clinical manifestation of rejection. Another study reported that individual changes in plasma BNP levels over time were more helpful than absolute BNP for detecting ISHLT grade 2R or greater rejection [41]. This further suggests that for BNP to be useful as a prognostic and diagnostic aid in cardiac allograft rejection, the history of BNP plasma values must be evaluated during follow-up visits.

In most studies reported to date, BNP plasma levels remained elevated for a relatively long time after cardiac allograft transplantation even without acute rejection episodes. A study found that in nonrejecting transplanted patients plasma BNP declined slowly following the transplant surgery without reaching the normal value during one-year observation [17, 42]. In another study it was shown that NT-proBNP levels in transplanted patients without complication continued to decrease for the 6 months after transplant [39]. The elevation in plasma BNP levels is not due to the surgical procedure. Plasma BNP levels after coronary artery bypass grafting, for example, return to near normal values one week after the surgery, while plasma BNP levels after heart transplant remain elevated for one week and did not return to normal values [18]. Cardiac allograft vasculopathy (CAV) could be related to these findings. As opposed to atherosclerosis, CAV is a diffuse and pan-arterial process, characterized by intimal thickening and luminal stenosis of coronary artery, which accounts for major morbidity and mortality [43]. Elevated plasma BNP levels were associated with CAV diagnosed by coronary angiography or by biopsy [31, 37, 44].

## 3. BNP and Cytokines

Because successful treatment of a rejection episode using the anti-T cell monoclonal antibody OKT3 decreased the elevated plasma BNP level to prerejection values [16], we hypothesized that cytokines activated during the inflammatory process may be involved in the observed increase in BNP blood levels during acute rejection. It was found that in neonatal rat ventricular cardiomyocytes (NRVCs) cultures, tumor necrosis factor alpha (TNF-*α*) and interleukin one beta (IL-1*β*), but not interferon gamma or interleukin 6, induced a significant increase in BNP mRNA and secretion, whereas ANF mRNA levels and secretion did not change. Inhibition of p38 MAP kinase signaling using SB203580 abolished IL-1*β*- and TNF-*α*-stimulated increase in BNP mRNA, promoter activity, and secretion. The conditioned medium obtained from unidirectional mixed lymphocyte reaction (MLR) culture, which is *in vitro* model of transplantation immunity, also increased BNP but not ANF secretion; an effect completely abolished by SB203580 pretreatment.

In the aforementioned study on transplant patients conducted in our laboratory, plasma IL-1*β* or TNF-*α* levels did not increase during acute rejection episodes, and further IL-1*β* and TNF-*α* blood levels did not correlate with BNP levels [17], nor does the conditioned medium from MLR cultures have detectable amounts of IL-1*β* or TNF-*α* suggesting that substances other than proinflammatory cytokines are responsible for the selective upregulation of BNP in inflammation. Nevertheless, studies of biopsy samples using RT-PCR or immunohistochemistry have demonstrated that IL-1*β* or TNF-*α* gene expression was increased during cardiac rejection suggesting that plasma levels of these cytokines do not reflect accurately the *in situ* level at the graft [45, 46]. To investigate whether other cytokines or factors in addition to TNF-*α* and IL-1*β* could be related to the upregulation of BNP during cardiac rejection, plasma samples taken from rejecting and nonrejecting patients were analyzed by cytokine array [47]. Regulated on activation, normal T expressed and secreted (RANTES), neutrophil activating protein-2 (NAP-2), and insulin growth factor binding protein-1 (IGFBP-1) plasma levels significantly correlate with BNP plasma levels during acute allograft rejection as diagnosed by endomyocardial biopsy. IGFBP-1 and RANTES induced BNP but not ANF secretion in NRVC cultures. In a separate study using 316 biopsy samples, the expression of RANTES measured by RT-PCR was found to increase significantly in ISHLT grades 2 and 3 rejection and returned to normal after treatment with pulse steroid therapy [48]. Another study, which measured the expression of 5000 genes in biopsy samples taken from 28 clinically quiescent patients, found that elevated plasma BNP was associated with genes related to cardiac structure remodeling, vascular injury, inflammation, and alloimmune processes [34].

## 4. BNP and the Immune System

Because successful treatment of an acute rejection episode with the anti-T cell antibody OKT3 decreased plasma BNP, we investigated the possibility that BNP may in turn have immunomodulatory properties on MLR cultures. Gene expression of the GC-A receptor in lymphocytes taken from MLR was significantly increased compared to that in the lymphocytes taken from individual Brown Norway or Lewis rats, which were not activated by MLR [49]. Addition of either ANF or BNP to the MLR cultures decreased cell proliferation suggesting that both ANF and BNP have immunomodulatory functions. Several observations from other laboratories are compatible with this notion. BNP is synthesized and secreted from macrophages and T cells infiltrating the failing myocardium [50] and was found to have immunomodulatory activity in human monocyte cell line as measured by the production of reactive oxygen and nitrogen species, leukotriene B_4_, prostaglandin E_2_, IL-10, and cell motility [51]. In addition, BNP directly induced apoptosis of CD8^+^ T cells taken from cardiac transplant patients via a caspase-3-associated mechanisms [52]. These studies suggest that even though both ANF and BNP can modulate proliferation of cell of the immune system, it is BNP that may carry out this function by virtue of the selective upregulation of this peptide by various neurohumoral species, including various cytokines and related substances involved in the immune response.

Studies investigating circulating BNP levels in rheumatoid arthritis (RA) patients have shown that NT-proBNP, C-reactive protein, and TNF-*α* levels in RA patients without clinical heart failure were higher than control groups suggesting that the increase in NT-proBNP in RA patients is related to inflammation [53]. Similarly, it has been reported that RA patients without history of cardiac disease had higher plasma BNP levels than a control group [54].

Taken together, all of the above studies link the endocrine function of the heart to the immune system.

## 5. BNP Gene Regulation at Transcriptional Level

In NRVC cultures, the upregulation of BNP gene expression caused by TNF-*α* and IL-1*β* can be prevented by actinomycin D, which suggests that the effect of cytokines on BNP gene expression is performed at transcriptional level. Further, NRVC transfected with −2.2-kb rat promoter coupled to a luciferase reporter gene, TNF-*α* and IL-1*β* stimulated the activation of −2.2-kb rat promoter, and SB203580, a p38 MAP kinase inhibitor, completely suppressed this effect [55]. In another study [56], mutation of the M-CAT element at −97-bp reduced IL-1*β* stimulation of the human BNP promoter activity by 60% suggesting that there are unknown elements which stimulate IL-1*β* activities. Mutation of other elements such as GATA at −85-bp and M-CAT at −124-bp did not affect IL-1*β* stimulation of the BNP promoter activity. Another proinflammatory cytokine, IL-6 also stimulated BNP synthesis and secretion in neonatal cardiomyocyte culture, and this elevation was inhibited by actinomycin D, but not cycloheximide, suggesting that this regulation is due to an increase in transcription [57].

## 6. BNP in Autoimmune Myocarditis

Immunization of rats with myosin heavy chain plus adjuvant produces an acute cardiac autoimmune response resembling giant cell myocarditis. In this myocarditis model, macrophages and CD4^+^ T cells are key components for the trigger of the disease [58]. In this model, 19 days after the immunization, TNF-*α* and IL-1*β* transcription are induced in ventricles and also heart weight/body weight ratio increases and mononuclear cells infiltration and necrosis are seen microscopically [59]. At this time we found that plasma BNP levels increase significantly in the absence of changes in ANF plasma levels in a manner reminiscent of what we previously observed in acute allograft rejection in humans suggesting that an increase in plasma BNP accompanied by unchanging circulating levels of ANF is indicative of inflammation *in vivo* [60].

## 7. BNP in Sepsis and following LPS Treatment

Several reports have indicated that plasma ANF or BNP levels are increased in sepsis and in septic shock patients [61–64]. The increase in plasma NPs is partly due to cardiac dysfunction because plasma ANF or BNP was inversely correlated with cardiac function [65–67]. However, in other reports where cardiac function was preserved, plasma BNP levels were elevated [61, 68, 69]. Administration of lipopolysaccharide (LPS) to healthy men increased plasma NT-proBNP without changing heart rate and blood pressure [70]. Also in the sepsis patients without systolic myocardial dysfunction, plasma BNP was correlated with C-reactive protein and IL-1*β* [61]. These findings are a further indication that elevated plasma BNP occurs in association with inflammation with or without cardiac dysfunction. In terms of a predictor of cardiac death in septic patients, plasma BNP was a better diagnostic parameter for the prediction of death than plasma ANF [71]. Hence, the changes in NP blood levels have been useful as predictors of outcomes. Plasma NT-proBNP, for example, was a predictor of nonsurvivors in severe sepsis patients [72].

To study the mechanism whereby plasma BNP changes in septic patients, we administered LPS—the main bacterial component responsible for the immune response to Gram-negative bacterial infection—to rats, and plasma and cardiac gene expression of NPs was investigated. Administration of LPS increased both plasma ANF and BNP levels although the increase in plasma BNP was always larger than the increase in ANF [73]. Gene expression of BNP was increased without changing the gene expression of ANF in both atria and ventricles. In this study, the correlation among mRNA data in 16 rats which included control, low-dose LPS, high-dose LPS, and LPS plus SB203580 rats were calculated, and it revealed that ANF mRNA was not correlated with BNP mRNA. The results also revealed that gene expression of MCP-1, MMP-8, TIMP-1 CINC-1, TNF-*α*, ICAM-1, and MIP-3 correlated with BNP gene expression although none of these mRNA levels correlated with ANF mRNA. These results confirm that BNP not ANF is related to the inflammation process.

## 8. The Effect of Inflammation on the Use of NPs as Biomarkers

While ANF and BNP are differently regulated in disorders involving inflammation such as in cardiac allograft rejection episode, sepsis, and experimental autoimmune myocarditis, these NPs are coordinately regulated in other cardiovascular diseases such as congestive heart failure, ischemic heart disease, and hypertensive heart diseases. In congestive heart failure plasma TNF-*α*, IL-1*β*, and IL-6 levels increase with the increase in New York Heart Association functional class [74]. The inflammation process is thus involved in most cardiovascular disorders together with hemodynamic and hormonal changes. The fact that BNP is more influenced by inflammation compared to ANF may be one reason why BNP appears as a marginally better biomarker for diagnostic and prognostic purposes in various cardiovascular disorders. Because inflammation and hemodynamic changes coexist in most cardiovascular disorders, the measurement of both ANF and BNP in individual patients may be advisable. Plasma ANF increases relatively larger than those of BNP may be indicative of hemodynamic deterioration, while the opposite observation may be indicative of worsening inflammation. Because these two pathologies are totally different and so are the indicated therapeutic interventions, measurement of ANF and BNP simultaneously may help to evaluate patient status more accurately. As a prognostic and diagnostic biomarker for cardiac allograft rejection in the individual patient, BNP plasma levels appear most useful when serially determined posttransplantation.
